# Sorafenib-Induced Erythema Multiforme Major and Severe Hepatic Failure in Metastatic Hepatocellular Carcinoma: A Case Report

**DOI:** 10.7759/cureus.57179

**Published:** 2024-03-29

**Authors:** Houda Abrini, Mounia Amzerin, Fatima Zahra El Mrabet

**Affiliations:** 1 Department of Medical Oncology, Mohammed VI University Hospital of Tangier, Faculty of Medicine and Pharmacy, Ahmed Bin Zayed Al Nahyan Center of Cancer Treatment, Abdelmalek Essaâdi University, Tangier, MAR

**Keywords:** case report, metastatic, erythema multiforme major, sorafenib, hepatocellular carcinoma

## Abstract

Sorafenib, a kinase inhibitor, is known to cause skin toxicity, which sometimes leads to treatment interruption or drug dose reduction. Erythema multiforme (EM) is one of these dermatologic toxicities induced by sorafenib. We report the case of a 28-year-old male with hepatocellular carcinoma (HCC). Two months after surgery, the patient presented with multiple metastases to the retroperitoneal lymph nodes and lungs. Therefore, systemic therapy with sorafenib was indicated. While receiving the medication, the patient presented signs compatible with EM. The signs occurred on the torso and then spread to the rest of the body. Sorafenib treatment was interrupted the same day when skin lesions appeared and moisturizers with topical steroids and oral antihistamines were prescribed. The skin lesions decreased in size but without significant cutaneous improvement. The patient showed biologically severe liver failure and radiological progression. Because of the severe hepatic failure, initiation of intravenous steroids and establishment of another line of chemotherapy following tumor progression were contraindicated. The decision of the multidisciplinary staff with patient consent was to proceed with the best supportive care. The patient died in ambulatory care 12 days after discharge and local treatment. This report highlights the possibility of developing severe EM while receiving sorafenib. Patients with HCC who have liver resection without liver dysfunction should not be administered sorafenib, or it must be used with caution at very low doses and accompanied by close and regular follow-ups to avoid disease progression and deaths.

## Introduction

Sorafenib is an oral multikinase inhibitor with antiproliferative and antiangiogenic activity [1]. Compared to best supportive care, it results in longer median overall survival rates when used as a first-line treatment in patients with advanced hepatocellular carcinoma (HCC) [2].

Skin toxicity is a common adverse effect of sorafenib and one of the leading causes of dose reduction or discontinuation of treatment [3]. Erythema multiforme (EM) induced by Sorafenib is a rare skin toxicity, and there is just one case report in the literature of EM that caused anaphylaxis in advanced HCC [3].

In this study, we report a singular observation of EM induced by sorafenib in a case of metastatic HCC preceded by hepatectomy.

## Case presentation

We present the case of a 28-year-old male patient with no previous record of viral hepatitis B or C, cirrhosis, or other comorbidities. Additionally, there was no history of medication use, allergies, or skin problems. Abdominal MRI confirmed an incidental finding by abdominal ultrasound of a liver lesion. The hepatic solid lesion measured 15.8 × 12 × 16.5 cm and was located between the hepatic segments VII and VIII. Moreover, the hepatic angio scan showed typical findings of HCC without metastasis in the chest scan. Imaging was compatible with Barcelona Clinic Liver Cancer (BCLC) early-stage A HCC.

The patient presented an Eastern Cooperative Oncology Group Performance Status (ECOG PS) scale of 0, alfa-fetoprotein level >2,000 ng/mL, Model of End-Stage Liver Disease (MELD) score of 6, and Child-Pugh class A. He underwent an extended right hepatectomy in segment IV. The histopathologic examination of the surgical specimen showed poorly differentiated HCC measuring 20 cm, a surgical limit located 0.2 cm from the tumor, and the presence of vascular emboli.

Two months after surgery, a chest scan and a hepatic angio scan were performed, demonstrating the appearance of multiple nodules in the hepatic left lobe, with typical findings of recurrent multifocal HCC, associated with multiple metastases of the lungs and retroperitoneal lymph node metastasis corresponding to the advanced stage (BCLC-C). The ECOG PS scale was 0-1. Systemic therapy with sorafenib 400 mg twice daily was indicated based on the decision of a multidisciplinary staff (immunotherapy was not feasible as it was not available).

**Figure 1:**
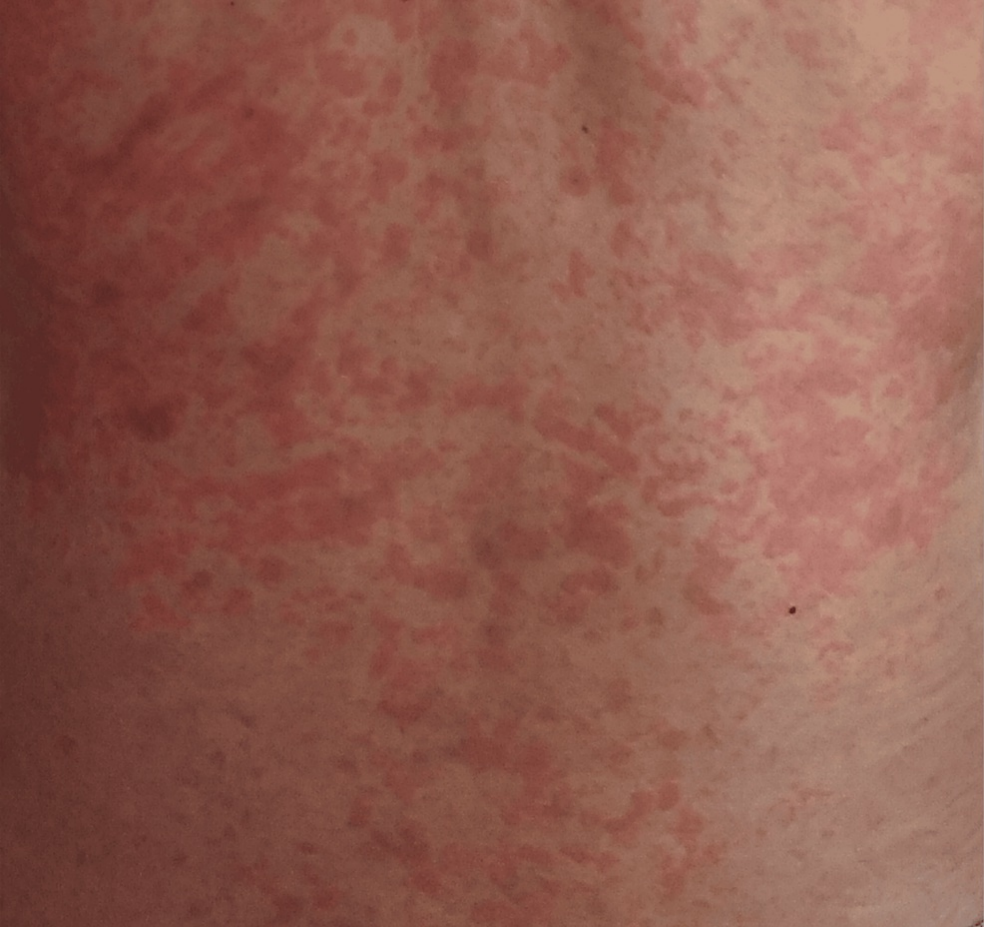
Erythema multiforme of the torso, abdomen, neck, and arms.

The initial biological examination was normal. Fifteen days after the start of sorafenib, the patient presented pruritic erythematous papules compatible with EM. Papules occurred initially on the thorax and then spread to the abdomen, neck, arms (Figure 1), legs, feet, and palmoplantar regions within three days. Painful oral aphthous ulcers and fever also appeared at the same time.

Sorafenib treatment was immediately discontinued and the patient was received in emergency medical care at our center and hospitalized on the same day. Upon admission, laboratory examination showed hepatic cytolysis: an increase in aspartate aminotransferase (AST) at 1,513 IU/L (previously 34 IU/L before starting sorafenib) and alanine aminotransferase (ALT) at 362 IU/L (previously 21 IU/L before starting sorafenib); hepatic cholestasis with total bilirubin of 134 mg/L, direct bilirubin of 74 mg/L, indirect bilirubin of 30 mg/L; decreased prothrombin rate at 38%; increased leukocytosis at 19,700/mm^3^; and important eosinophilia at 611/mm^3^ (Table 1).

**Table 1:** Liver function tests of the patient.

Liver function tests	Reference range	Initial (before treatment)	Upon admission	Fourth day of hospitalisation
Aspartate aminotransferase (IU/L)	<35	34	1,513	5,167
Alanine aminotransferase (IU/L)	<35	21	362	796
Total bilirubin (mg/L)	0–13	13	134	192
Direct bilirubin (mg/L)	0–3	3	74	183
Indirect bilirubine (mg/L)	0–10	10	30	47
Prothrombin rate (%)	70–100	100	38	33

The skin lesions were diagnosed as grade 4 CTCAEv5.0 (Common Terminology Criteria for Adverse Events) and the diagnosis was EM major induced by sorafenib. The patient was prescribed moisturizers with topical steroids and oral antihistamines 5 mg daily by the dermatologist. As a result, the lesions decreased in size but without significant cutaneous improvement.

**Figure 2:**
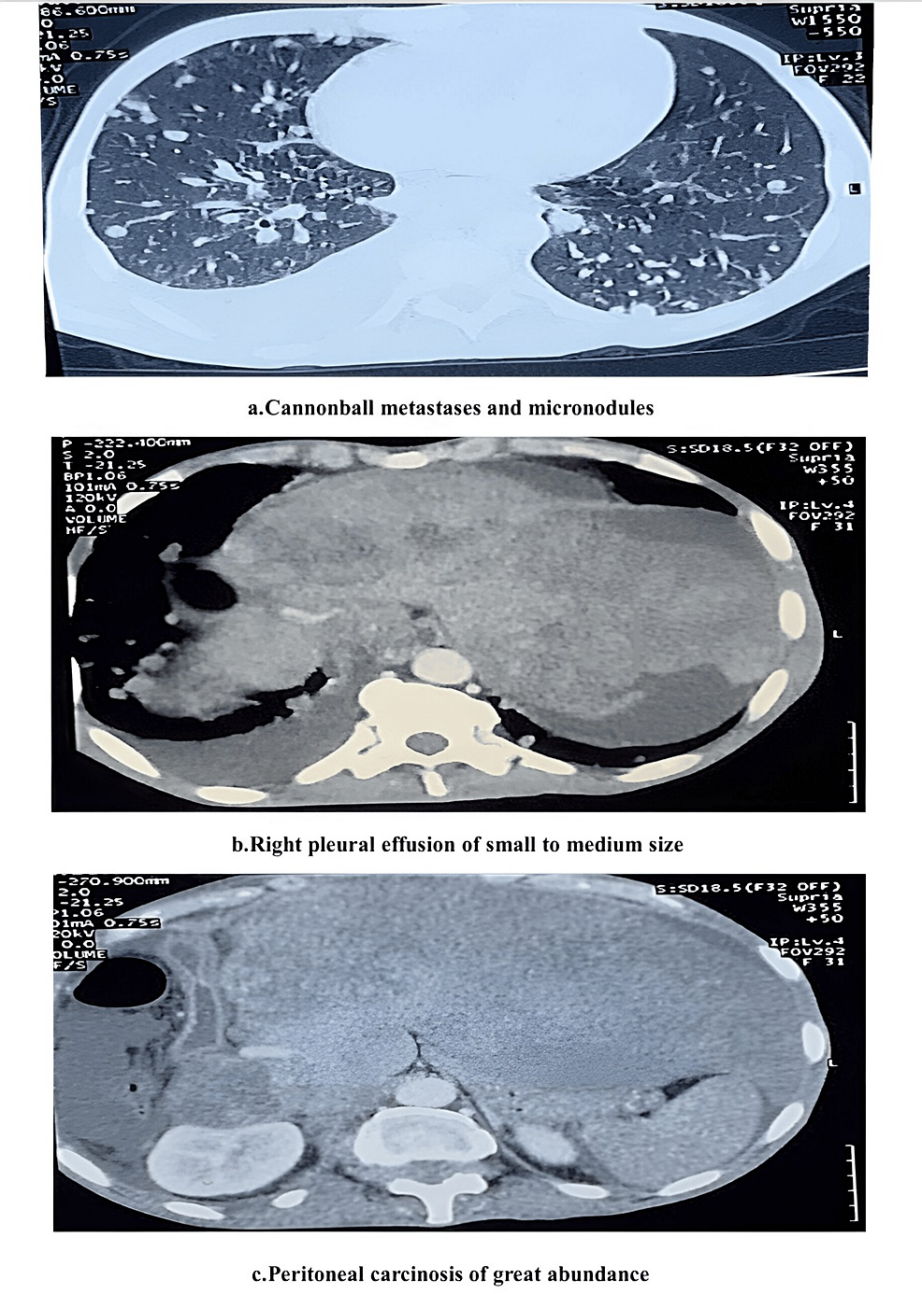
Control scan 18 days after the start of sorafenib.

Eighteen days after the start of sorafenib treatment, a control scan showed radiological progression as follows (Figure 2): cannonball metastases and micronodules (a), with pleural effusion of small to medium size (b), large mediastinal lymph node metastasis, thrombosis of portal vein and inferior vein cava, and peritoneal carcinosis of great abundance (c).

Because of the severe hepatic failure (terminal stage, BCLC-D; Table 1) during his hospitalization (AST = 5,167 IU/L and ALT = 796 IU/L, total bilirubin = 192 mg/L, direct bilirubin = 183 mg/L, indirect bilirubin = 47 mg/L and prothrombin = 33%), initiation of intravenous steroids and establishment of another line of chemotherapy following tumor progression were contraindicated. The decision of the multidisciplinary staff with patient consent was to proceed with the best supportive care to manage symptoms quickly, including pain treatment, transfusion of blood products, treating infections with antibiotics, and continuing topical steroids and oral antihistamines to provide the best quality of life. Twelve days after discharge and local treatment, the patient died in ambulatory care.

## Discussion

In our case report, the patient presented with HCC (BCLC-C). Therefore, systemic therapy with sorafenib was indicated. Treatment with immunotherapy (atezolizumab or durvalumab) was not feasible because of its inaccessibility. According to Reig et al., in the advanced stage (BCLC-C), sorafenib can be proposed as an option, as it is associated with improved overall survival [4].

Sorafenib-induced EM reportedly occurs in only 0.1%-1% of patients [5]. The patient in our case report did not have a biopsy. Indeed, there are numerous published reports of sorafenib-induced EM, but most of these cases did not report histopathologic confirmation. To avoid unnecessary discontinuation of sorafenib, a biopsy is recommended for any suspected EM lesions [6].

EM major appeared in our patient on the 15th day of sorafenib treatment. The drug was discontinued immediately, and the patient was treated with topical steroids and oral antihistamines without oral or intravenous corticosteroids. Wan et al. found that oral prednisone was not beneficial in severe drug-induced liver injury [7]. Indeed, corticosteroids have not demonstrated an improvement in overall survival in drug-induced acute liver failure and have shown a low survival in high MELD patients [8].

Previous studies have reported the appearance of rashes within the first days or weeks of treatment. These rashes typically only necessitate the use of antihistamines and topical steroids for relief, without any alterations or interruptions in the sorafenib regimen [9]. In another study, the authors concluded that patients who experienced skin toxicity/diarrhea while receiving sorafenib at or close doses to the phase II/III trials (RDP) gained significantly more benefit than those without toxicity [10]. Hence, the interruption of treatment is not necessarily the right decision [10].

Sorafenib was suspended upon the onset of the severe skin toxicity in our case. The disease progressed, and the patient died 12 days after drug interruption. According to BCLC recommendations, treatment should be interrupted for severe adverse events (grade 3/4) [11]. If the adverse events return to baseline status, the accountability of sorafenib is confirmed and the maximum tolerable dose should be defined. If the adverse events do not return to baseline status, tumor progression, cirrhosis complications, and other etiologies should be ruled out [12].

Our patient had developed severe hepatic failure (terminal stage, BCLC-D). Most patients with HCC have underlying liver disease, and local treatment with sorafenib should be applied with caution because it may increase the risk of severe hepatotoxicity. Patients treated with sorafenib require dose reduction after interventional treatment [13].

A Japanese study in which 70% of patients were classified as BCLC-C with 52% extrahepatic metastases and 93% were treated with surgery, local ablation, or transarterial chemoembolization before receiving sorafenib therapy demonstrated that the majority of patients needed a reduction of sorafenib dose and showed that adverse events, including rash and liver failure, occurred with higher rates. Close monitoring should be done to avoid serious adverse events during the administration of sorafenib [14].

## Conclusions

We concur that the EM lesion should be biopsied to confirm its histology and sorafenib should be permanently discontinued if the diagnosis of EM major is confirmed. We aim to emphasize that patients with HCC who have liver resection without liver dysfunction should either not be treated with sorafenib, or the drug should be administrated with caution at very low doses to identify and limit EM major and avoid disease progression and deaths. Therefore, the risk-benefit ratio of sorafenib should be discussed and taken into consideration. Finally, further investigations are needed to disclose the molecular mechanisms involved in sorafenib toxicity and to better characterize EM major induced by sorafenib.
